# A novel approach to target metastases of melanoma cells in an organ-selective manner

**DOI:** 10.1186/1479-5876-12-S1-P2

**Published:** 2014-05-06

**Authors:** Saurav Roy Choudhury, Jennifer Rahn, Xiaoguang Hao, Liane Babes, Paul Kubes, Stephen M Robbins, Donna L Senger

**Affiliations:** 1 Department of Oncology, University of Calgary and Southern Alberta Cancer Research Institute, Calgary, Canada; 2 Department of Biochemistry and Molecular Biology, University of Calgary, Calgary, Canada; 3 Snyder Institute for Chronic Diseases, Department of Physiology and Pharmacology, University of Calgary, Calgary, Canada, T2N 4N1

## Background

Cancer is a leading cause of death globally and the majority of patients will die from the formation of metastases rather than from their primary tumour as most metastases are resistant to conventional therapies. Based on numerous clinical observations it is clear that different cancers have a propensity to metastasize to specific organs. The central hypothesis for our current study is that an organs vasculature is intimately involved in mediating the interaction between specific cancer cells and the target organ for which they will metastasize to. In this respect we have focused on how melanoma cancer cells metastasize to the liver and lungs, two major sites of metastatic disease within the body, in order to provide insight for the development of new treatments for metastatic cancer.

## Materials and methods

Herein we used an unbiased combinatorial phage in vivo biopanning strategy to isolate a library of peptide-displaying-phage that home to the liver and lungs of animals treated with a pro-inflammatory stimulus, lipopolysccharaide (LPS). Using a functional screen we isolated a single phage that prevented the recruitment of leukocytes into the liver and lungs of animals following LPS treatment. We investigated if the lung/liver inhibitory phage were also able to inhibit the formation of lung metastasis using a human melanoma metastasis xenograft model.

## Results

Using intravital microscopy, we identified a peptide-displaying-phage, and its corresponding displayed-peptide, that has the ability to inhibit adhesion and recruitment of neutrophils in the liver sinusoids in response to LPS. Excitingly, we also found that animals injected (i.v.) with human melanoma cells (70W) in the presence of the phage, or its corresponding displayed-peptide, showed reduced metastatic lung burden. As the isolated peptide was capable of inhibiting both leukocyte recruitment and melanoma lung metastasis, we asked if leukocytes played a functional role in the establishment of melanoma metastasis. We found that in animals that received Lys6G (Anti Gr-1) antibody to deplete circulating leukocytes there was a dramatic increase in 70W melanoma metastasis to the lungs and that this increase in metastatic state was still blocked by the addition of the peptide.

## Conclusion

These results suggest two major findings, first that circulating leukocytes could have a protective role in preventing melanoma metastasis to the lungs and second that the peptide identified in this study may work by blocking molecules that are required for recruitment and/or extravasation of the cancer cells, a process that is shared by leukocytes.

